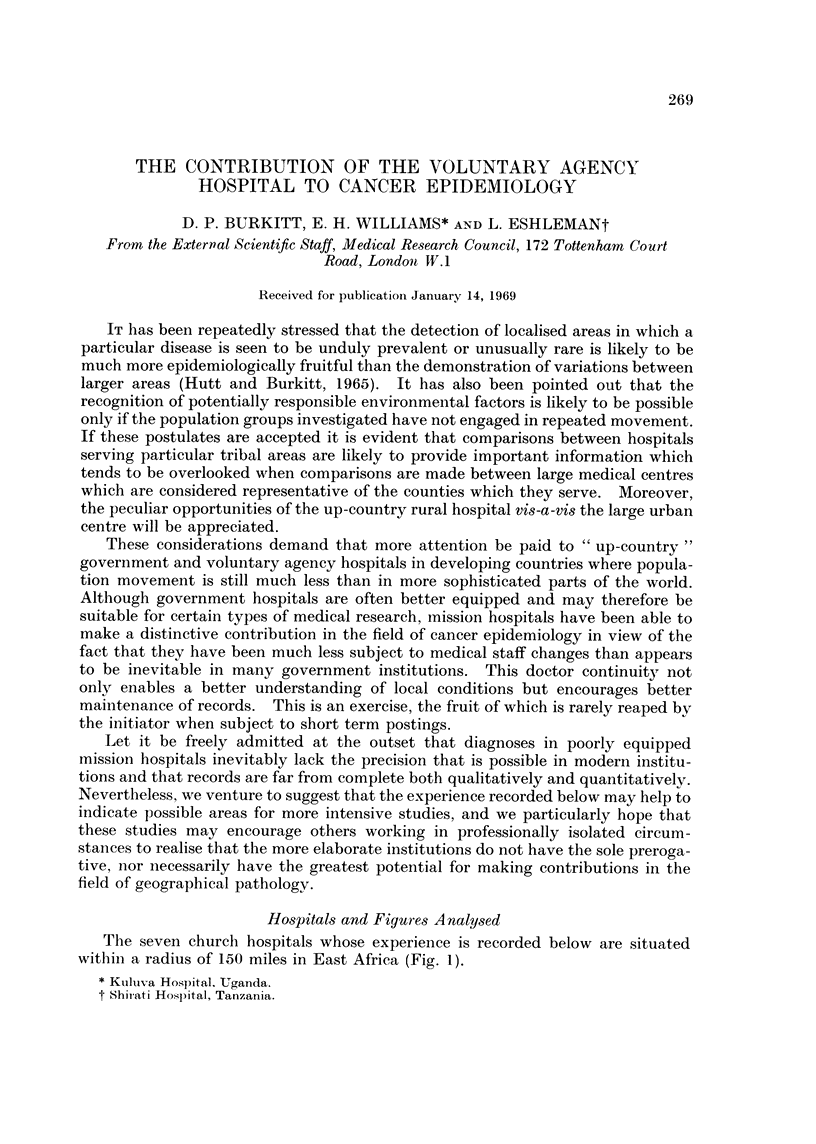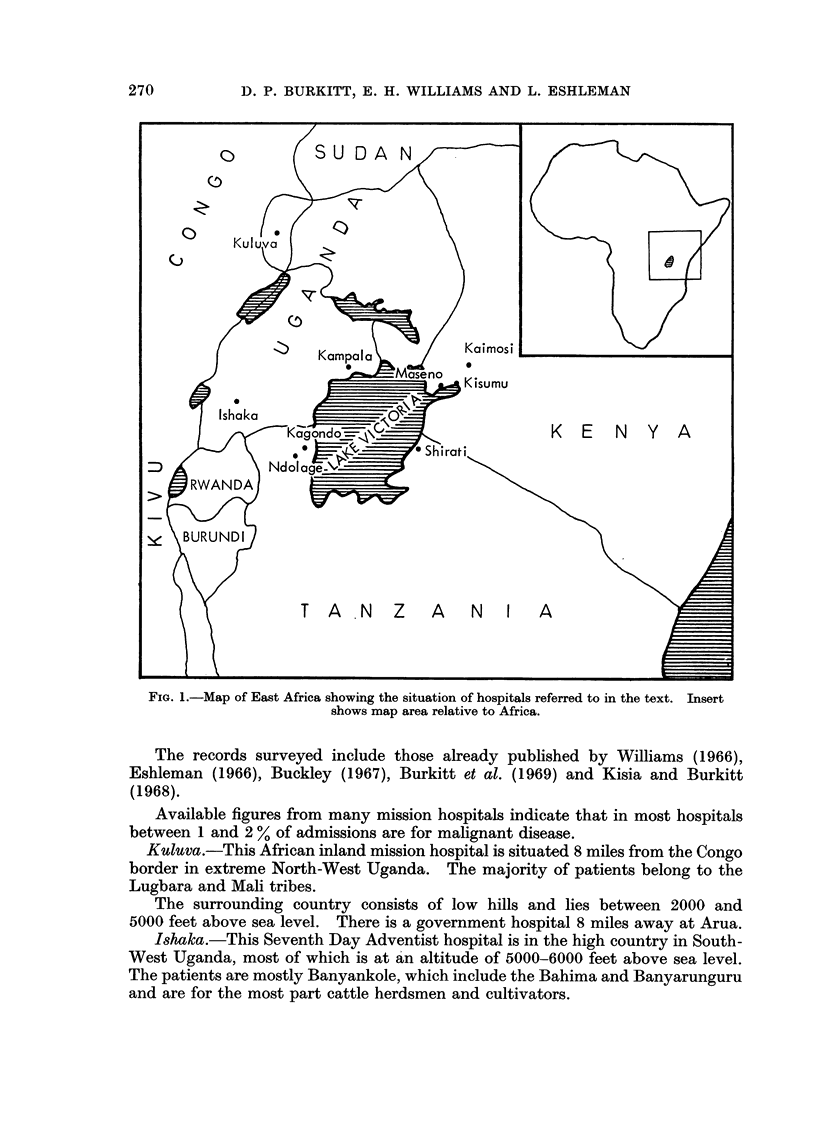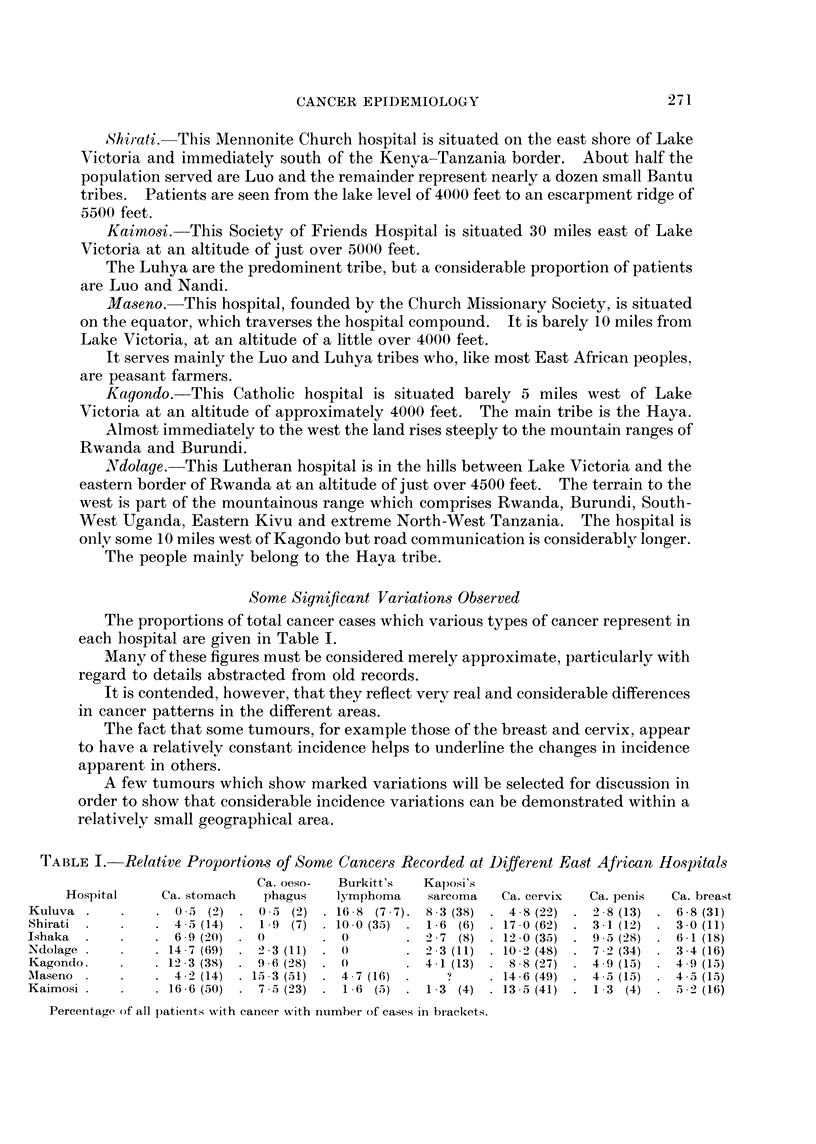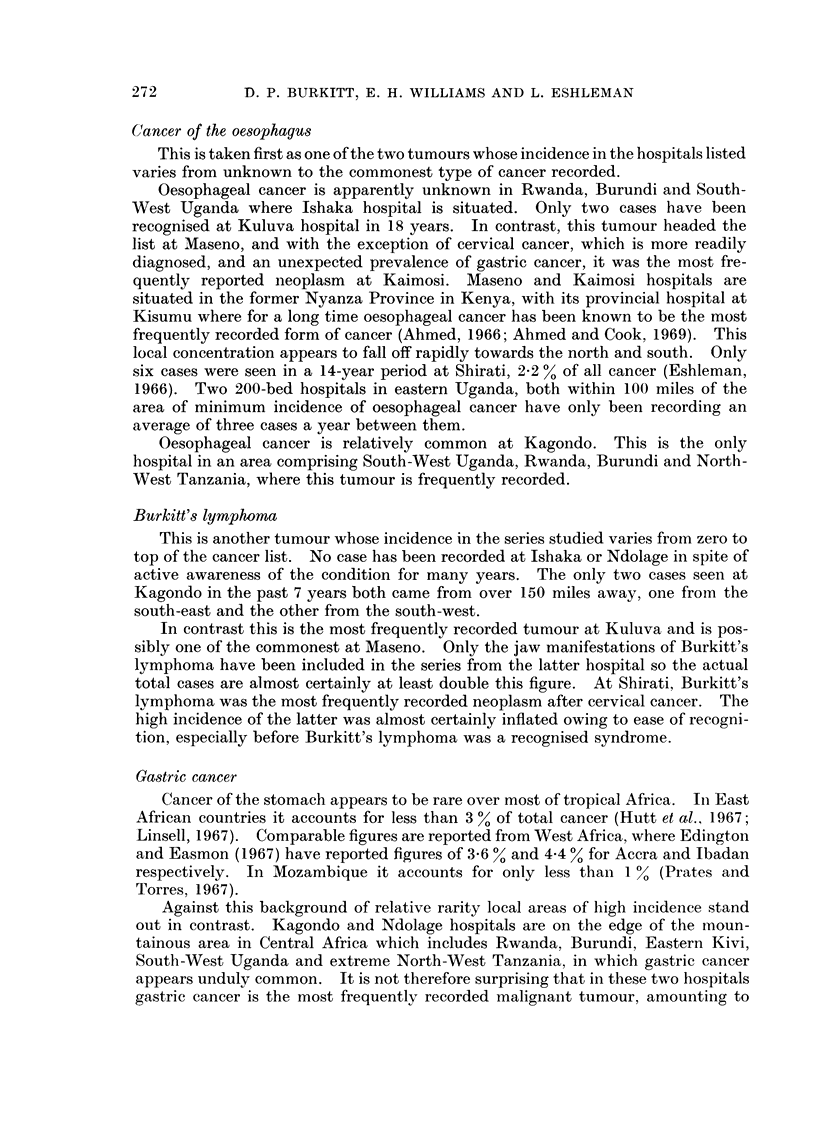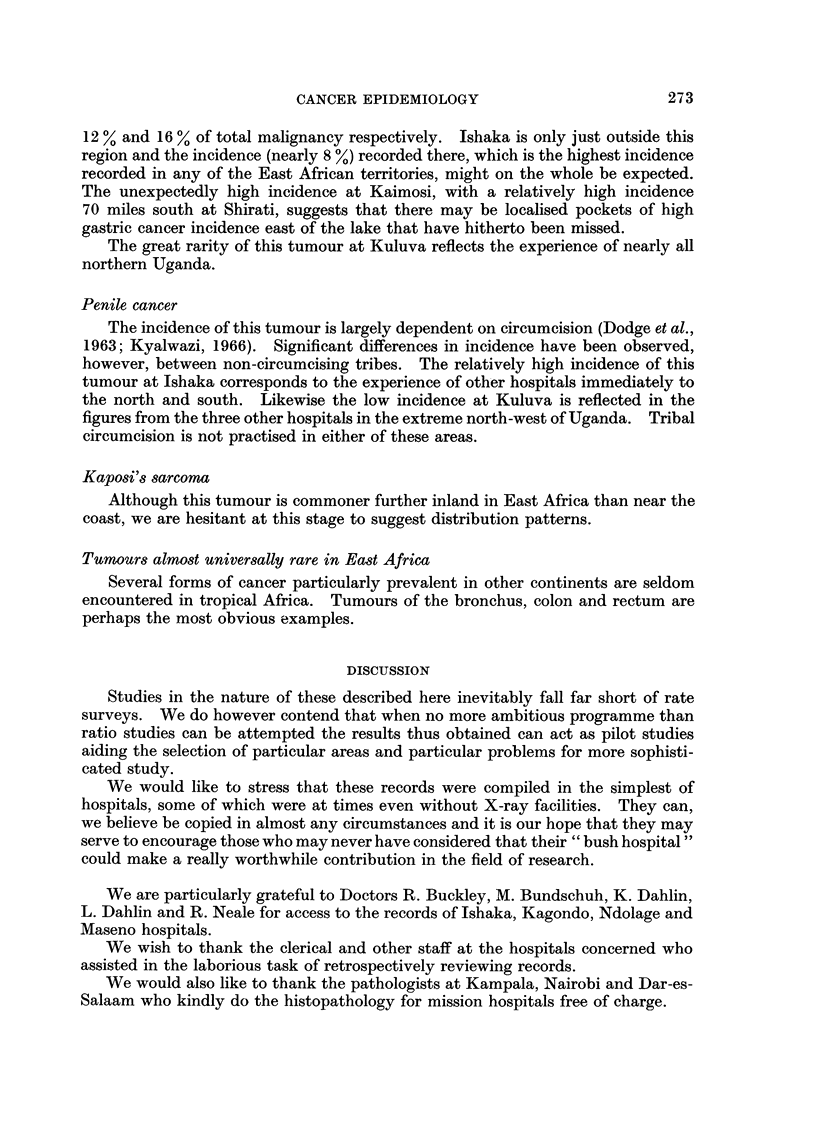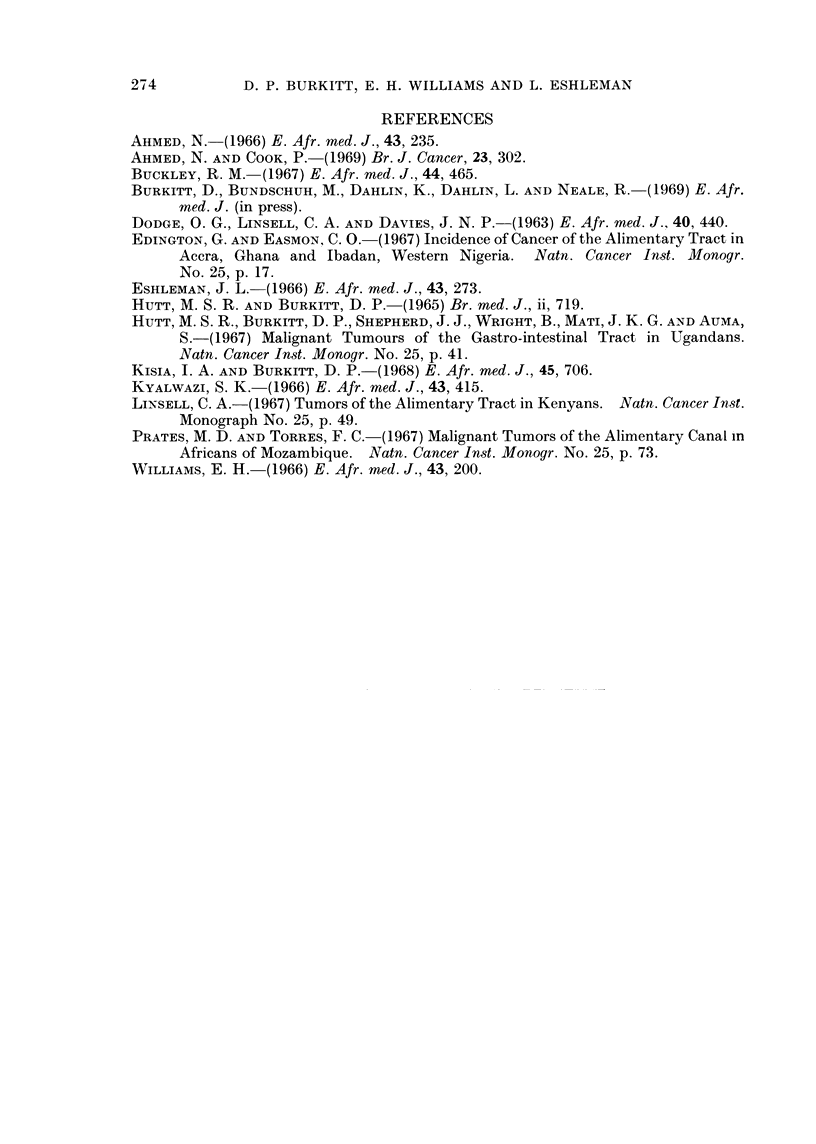# The contribution of the voluntary agency hospital to cancer epidemiology.

**DOI:** 10.1038/bjc.1969.36

**Published:** 1969-06

**Authors:** D. P. Burkitt, E. H. Williams, L. Eshleman


### THE CONTRIBUTION OF THE VOLUNTARY AGENCY HOSPITAL TO CANCER EPIDEMIOLOGY

D. P. BURKITT, E. H. WILLIAMS* AND L. ESHLEMAN†

*From the External Scientific Staff, Medical Research Council,* 172 *Tottenham Court Road, London W.*1

Received for publication January 14, 1969

It has been repeatedly stressed that the detection of localised areas in which a particular disease is seen to be unduly prevalent or unusually rare is likely to be much more epidemiologically fruitful than the demonstration of variations between larger areas (Hutt and Burkitt, 1965). It has also been pointed out that the recognition of potentially responsible environmental factors is likely to be possible only if the population groups investigated have not engaged in repeated movement. If these postulates are accepted it is evident that comparisons between hospitals serving particular tribal areas are likely to provide important information which tends to be overlooked when comparisons are made between large medical centres which are considered representative of the counties which they serve. Moreover, the peculiar opportunities of the up-country rural hospital *vis-a-vis* the large urban centre will be appreciated.

These considerations demand that more attention be paid to " up-country " government and voluntary agency hospitals in developing countries where population movement is still much less than in more sophisticated parts of the world. Although government hospitals are often better equipped and may therefore be suitable for certain types of medical research, mission hospitals have been able to make a distinctive contribution in the field of cancer epidemiology in view of the fact that they have been much less subject to medical staff changes than appears to be inevitable in many government institutions. This doctor continuity not only enables a better understanding of local conditions but encourages better maintenance of records. This is an exercise, the fruit of which is rarely reaped by the initiator when subject to short term postings.

Let it be freely admitted at the outset that diagnoses in poorly equipped mission hospitals inevitably lack the precision that is possible in modern institutions and that records are far from complete both qualitatively and quantitatively. Nevertheless, we venture to suggest that the experience recorded below may help to indicate possible areas for more intensive studies, and we particularly hope that these studies may encourage others working in professionally isolated circumstances to realise that the more elaborate institutions do not have the sole prerogative, nor necessarily have the greatest potential for making contributions in the field of geographical pathology.

### *Hospitals and Figures Analysed*

The seven church hospitals whose experience is recorded below are situated within a radius of 150 miles in East Africa (Fig. 1).

* Kuluva Hospital, Uganda.
† Shirati Hospital, Tanzania.

FIG. 1.—Map of East Africa showing the situation of hospitals referred to in the text. Insert shows map area relative to Africa.

The records surveyed include those already published by Williams (1966), Eshleman (1966), Buckley (1967), Burkitt *et al.* (1969) and Kisia and Burkitt (1968).

Available figures from many mission hospitals indicate that in most hospitals between 1 and 2 % of admissions are for malignant disease.

*Kuluva.*—This African inland mission hospital is situated 8 miles from the Congo border in extreme North-West Uganda. The majority of patients belong to the Lugbara and Mali tribes.

The surrounding country consists of low hills and lies between 2000 and 5000 feet above sea level. There is a government hospital 8 miles away at Arua.

*Ishaka.*—This Seventh Day Adventist hospital is in the high country in South-West Uganda, most of which is at an altitude of 5000–6000 feet above sea level. The patients are mostly Banyankole, which include the Bahima and Banyarunguru and are for the most part cattle herdsmen and cultivators.

*Shirati*.—This Mennonite Church hospital is situated on the east shore of Lake Victoria and immediately south of the Kenya–Tanzania border. About half the population served are Luo and the remainder represent nearly a dozen small Bantu tribes. Patients are seen from the lake level of 4000 feet to an escarpment ridge of 5500 feet.

*Kaimosi*.—This Society of Friends Hospital is situated 30 miles east of Lake Victoria at an altitude of just over 5000 feet.

The Luhya are the predominent tribe, but a considerable proportion of patients are Luo and Nandi.

*Maseno*.—This hospital, founded by the Church Missionary Society, is situated on the equator, which traverses the hospital compound. It is barely 10 miles from Lake Victoria, at an altitude of a little over 4000 feet.

It serves mainly the Luo and Luhya tribes who, like most East African peoples, are peasant farmers.

*Kagondo*.—This Catholic hospital is situated barely 5 miles west of Lake Victoria at an altitude of approximately 4000 feet. The main tribe is the Haya.

Almost immediately to the west the land rises steeply to the mountain ranges of Rwanda and Burundi.

*Ndolage*.—This Lutheran hospital is in the hills between Lake Victoria and the eastern border of Rwanda at an altitude of just over 4500 feet. The terrain to the west is part of the mountainous range which comprises Rwanda, Burundi, South-West Uganda, Eastern Kivu and extreme North-West Tanzania. The hospital is only some 10 miles west of Kagondo but road communication is considerably longer.

The people mainly belong to the Haya tribe.

### *Some Significant Variations Observed*

The proportions of total cancer cases which various types of cancer represent in each hospital are given in Table I.

Many of these figures must be considered merely approximate, particularly with regard to details abstracted from old records.

It is contended, however, that they reflect very real and considerable differences in cancer patterns in the different areas.

The fact that some tumours, for example those of the breast and cervix, appear to have a relatively constant incidence helps to underline the changes in incidence apparent in others.

A few tumours which show marked variations will be selected for discussion in order to show that considerable incidence variations can be demonstrated within a relatively small geographical area.

TABLE I.—*Relative Proportions of Some Cancers Recorded at Different East African Hospitals*

| Hospital | Ca. stomach | Ca. oesophagus | Burkitt's lymphoma | Kaposi's sarcoma | Ca. cervix | Ca. penis | Ca. breast |
|---|---|---|---|---|---|---|---|
| Kuluva | 0·5 (2) | 0·5 (2) | 16·8 (7·7) | 8·3 (38) | 4·8 (22) | 2·8 (13) | 6·8 (31) |
| Shirati | 4·5 (14) | 1·9 (7) | 10·0 (35) | 1·6 (6) | 17·0 (62) | 3·1 (12) | 3·0 (11) |
| Ishaka | 6·9 (20) | 0 | 0 | 2·7 (8) | 12·0 (35) | 9·5 (28) | 6·1 (18) |
| Ndolage | 14·7 (69) | 2·3 (11) | 0 | 2·3 (11) | 10·2 (48) | 7·2 (34) | 3·4 (16) |
| Kagondo | 12·3 (38) | 9·6 (28) | 0 | 4·1 (13) | 8·8 (27) | 4·9 (15) | 4·9 (15) |
| Maseno | 4·2 (14) | 15·3 (51) | 4·7 (16) | ? | 14·6 (49) | 4·5 (15) | 4·5 (15) |
| Kaimosi | 16·6 (50) | 7·5 (23) | 1·6 (5) | 1·3 (4) | 13·5 (41) | 1·3 (4) | 5·2 (16) |

Percentage of all patients with cancer with number of cases in brackets.

*Cancer of the oesophagus*

This is taken first as one of the two tumours whose incidence in the hospitals listed varies from unknown to the commonest type of cancer recorded.

Oesophageal cancer is apparently unknown in Rwanda, Burundi and South-West Uganda where Ishaka hospital is situated. Only two cases have been recognised at Kuluva hospital in 18 years. In contrast, this tumour headed the list at Maseno, and with the exception of cervical cancer, which is more readily diagnosed, and an unexpected prevalence of gastric cancer, it was the most frequently reported neoplasm at Kaimosi. Maseno and Kaimosi hospitals are situated in the former Nyanza Province in Kenya, with its provincial hospital at Kisumu where for a long time oesophageal cancer has been known to be the most frequently recorded form of cancer (Ahmed, 1966; Ahmed and Cook, 1969). This local concentration appears to fall off rapidly towards the north and south. Only six cases were seen in a 14-year period at Shirati, 2·2 % of all cancer (Eshleman, 1966). Two 200-bed hospitals in eastern Uganda, both within 100 miles of the area of minimum incidence of oesophageal cancer have only been recording an average of three cases a year between them.

Oesophageal cancer is relatively common at Kagondo. This is the only hospital in an area comprising South-West Uganda, Rwanda, Burundi and North-West Tanzania, where this tumour is frequently recorded.

*Burkitt's lymphoma*

This is another tumour whose incidence in the series studied varies from zero to top of the cancer list. No case has been recorded at Ishaka or Ndolage in spite of active awareness of the condition for many years. The only two cases seen at Kagondo in the past 7 years both came from over 150 miles away, one from the south-east and the other from the south-west.

In contrast this is the most frequently recorded tumour at Kuluva and is possibly one of the commonest at Maseno. Only the jaw manifestations of Burkitt's lymphoma have been included in the series from the latter hospital so the actual total cases are almost certainly at least double this figure. At Shirati, Burkitt's lymphoma was the most frequently recorded neoplasm after cervical cancer. The high incidence of the latter was almost certainly inflated owing to ease of recognition, especially before Burkitt's lymphoma was a recognised syndrome.

*Gastric cancer*

Cancer of the stomach appears to be rare over most of tropical Africa. In East African countries it accounts for less than 3 % of total cancer (Hutt *et al.*, 1967; Linsell, 1967). Comparable figures are reported from West Africa, where Edington and Easmon (1967) have reported figures of 3·6 % and 4·4 % for Accra and Ibadan respectively. In Mozambique it accounts for only less than 1 % (Prates and Torres, 1967).

Against this background of relative rarity local areas of high incidence stand out in contrast. Kagondo and Ndolage hospitals are on the edge of the mountainous area in Central Africa which includes Rwanda, Burundi, Eastern Kivi, South-West Uganda and extreme North-West Tanzania, in which gastric cancer appears unduly common. It is not therefore surprising that in these two hospitals gastric cancer is the most frequently recorded malignant tumour, amounting to

12 % and 16 % of total malignancy respectively. Ishaka is only just outside this region and the incidence (nearly 8 %) recorded there, which is the highest incidence recorded in any of the East African territories, might on the whole be expected. The unexpectedly high incidence at Kaimosi, with a relatively high incidence 70 miles south at Shirati, suggests that there may be localised pockets of high gastric cancer incidence east of the lake that have hitherto been missed.

The great rarity of this tumour at Kuluva reflects the experience of nearly all northern Uganda.

*Penile cancer*

The incidence of this tumour is largely dependent on circumcision (Dodge *et al.*, 1963; Kyalwazi, 1966). Significant differences in incidence have been observed, however, between non-circumcising tribes. The relatively high incidence of this tumour at Ishaka corresponds to the experience of other hospitals immediately to the north and south. Likewise the low incidence at Kuluva is reflected in the figures from the three other hospitals in the extreme north-west of Uganda. Tribal circumcision is not practised in either of these areas.

*Kaposi's sarcoma*

Although this tumour is commoner further inland in East Africa than near the coast, we are hesitant at this stage to suggest distribution patterns.

*Tumours almost universally rare in East Africa*

Several forms of cancer particularly prevalent in other continents are seldom encountered in tropical Africa. Tumours of the bronchus, colon and rectum are perhaps the most obvious examples.

### DISCUSSION

Studies in the nature of these described here inevitably fall far short of rate surveys. We do however contend that when no more ambitious programme than ratio studies can be attempted the results thus obtained can act as pilot studies aiding the selection of particular areas and particular problems for more sophisticated study.

We would like to stress that these records were compiled in the simplest of hospitals, some of which were at times even without X-ray facilities. They can, we believe be copied in almost any circumstances and it is our hope that they may serve to encourage those who may never have considered that their "bush hospital" could make a really worthwhile contribution in the field of research.

We are particularly grateful to Doctors R. Buckley, M. Bundschuh, K. Dahlin, L. Dahlin and R. Neale for access to the records of Ishaka, Kagondo, Ndolage and Maseno hospitals.

We wish to thank the clerical and other staff at the hospitals concerned who assisted in the laborious task of retrospectively reviewing records.

We would also like to thank the pathologists at Kampala, Nairobi and Dar-es-Salaam who kindly do the histopathology for mission hospitals free of charge.

### REFERENCES

AHMED, N.—(1966) *E. Afr. med. J.*, **43**, 235.

AHMED, N. AND COOK, P.—(1969) *Br. J. Cancer*, **23**, 302.

BUCKLEY, R. M.—(1967) *E. Afr. med. J.*, **44**, 465.

BURKITT, D., BUNDSCHUH, M., DAHLIN, K., DAHLIN, L. AND NEALE, R.—(1969) *E. Afr. med. J.* (in press).

DODGE, O. G., LINSELL, C. A. AND DAVIES, J. N. P.—(1963) *E. Afr. med. J.*, **40**, 440.

EDINGTON, G. AND EASMON, C. O.—(1967) Incidence of Cancer of the Alimentary Tract in Accra, Ghana and Ibadan, Western Nigeria. *Natn. Cancer Inst. Monogr.* No. 25, p. 17.

ESHLEMAN, J. L.—(1966) *E. Afr. med. J.*, **43**, 273.

HUTT, M. S. R. AND BURKITT, D. P.—(1965) *Br. med. J.*, ii, 719.

HUTT, M. S. R., BURKITT, D. P., SHEPHERD, J. J., WRIGHT, B., MATI, J. K. G. AND AUMA, S.—(1967) Malignant Tumours of the Gastro-intestinal Tract in Ugandans. *Natn. Cancer Inst. Monogr.* No. 25, p. 41.

KISIA, I. A. AND BURKITT, D. P.—(1968) *E. Afr. med. J.*, **45**, 706.

KYALWAZI, S. K.—(1966) *E. Afr. med. J.*, **43**, 415.

LINSELL, C. A.—(1967) Tumors of the Alimentary Tract in Kenyans. *Natn. Cancer Inst.* Monograph No. 25, p. 49.

PRATES, M. D. AND TORRES, F. C.—(1967) Malignant Tumors of the Alimentary Canal in Africans of Mozambique. *Natn. Cancer Inst. Monogr.* No. 25, p. 73.

WILLIAMS, E. H.—(1966) *E. Afr. med. J.*, **43**, 200.